# Engineering of Therapeutic Proteins Production in *Escherichia coli*

**DOI:** 10.2174/138920111794295693

**Published:** 2011-02

**Authors:** Mariusz Kamionka

**Affiliations:** School of Pharmacy and Pharmaceutical Sciences, Trinity College, University of Dublin, Dublin 2, Ireland

**Keywords:** Biopharmaceuticals, drug development, protein drugs, protein engineering, protein expression, therapeutic proteins.

## Abstract

Low cost and simplicity of cultivating bacteria make the *E. coli* expression system a preferable choice for production of therapeutic proteins both on a lab scale and in industry. In addition straightforward recombinant DNA technology offers engineering tools to produce protein molecules with modified features. The lack of posttranslational modification mechanisms in bacterial cells such as glycosylation, proteolytic protein maturation or limited capacity for formation of disulfide bridges may, to a certain extent, be overcome with protein engineering. Protein engineering is also often employed to improve protein stability or to modulate its biological action. More sophisticated modifications may be achieved by genetic fusions of two proteins. This article presents a variety of examples of genetic engineering of therapeutic proteins. It emphasizes the importance of designing a construct without any unnecessary amino acid residues.

## INTRODUCTION

Many years have passed since recombinant human insulin, the first medicine made via recombinant DNA technology, was approved by the FDA [1]. Today we are witnessing the continuous rise in the number of approved protein therapeutics [2] and there is little doubt that biopharmaceuticals have the potential to become the medicines of the future. 

Recombinant DNA technology not only allows therapeutic proteins to be produced on a large scale but using the same methodology protein molecules may be purposefully engineered (Table **1**). Genetic modifications introduced to a protein have many advantages over chemical modifications. Genetically engineered entities are biocompatible and biodegradable. The changes are introduced in 100% of the molecules with the exclusion of rare errors in gene transcription or translation. The preparations do not contain residual amounts of harsh chemicals used in the conjugation process. 

Bacterial expression systems, due to their simplicity, are often not able to produce a recombinant human protein identical to the naturally occurring wild type. Bacteria did not develop sophisticated mechanisms for performing posttranslational modifications which are present in higher organisms. As a consequence, an increasing number of protein therapeutics is expressed in mammalian cells. However the low cost and simplicity of cultivating bacteria is an unbeatable advantage over any other expression system and therefore *E. coli* is always a preferable choice both on a lab scale and in industry. 

The lack of some posttranslational modifications in bacterial cells may, to a certain extent, be overcome with protein engineering and this is the focus of the first part of this article. Protein engineering may be employed for other purposes as well, and the second part of the article presents selected examples of attempts to improve protein stability or to modulate its biological action. It also presents some applications where two proteins were genetically fused together. The importance of a “clean” construct without any unnecessary amino acid residues is also discussed.

The number of approved protein therapeutics is constantly growing, and the most dynamic subgroup is monoclonal antibodies. However due to the complexity of their molecules they can only be produced in eukaryotic expression systems and therefore they are not covered in this review.

## OVERCOMING MISSING POSTTRANSLATIONAL MODIFICATIONS

The amino acid sequence of a recombinant human protein expressed in *E. coli* may be identical to the human wild type and yet the protein may lack or have a decreased biological activity. Bacteria, being an example of prokaryotic organisms, differ from the higher (eukaryotic) organisms in that they are not able to perform the majority of protein posttranslational modifications for which their cell would require specialized cell compartments.

### Glycosylation

One of the major types of posttranslational modifications absent in *E. coli* is glycosylation. The biological roles of glycans added to a protein in the glycosylation process span the spectrum, from those that appear to be relatively subtle, to those that are crucial for the development, growth, functioning, or survival of the organism that synthesizes them [21]. Obviously these proteins for which the glycosylation plays an important role in their biological activity must be expressed in mammalian cells. However there are glycoproteins which do not require glycans to exert their function. In such cases the *E. coli* expression system may be successfully employed. It was shown for instance that the recombinant human interleukin-2 (IL-2) produced in *E. coli* has the same biological activity as the glycosylated IL-2 version isolated from cultured mammalian cells ^3^. Indeed aldesleukin (recombinant IL-2) which was marketed by Chiron Corporation (part of Novartis since April 2006) to treat patients with metastatic melanoma under the brand name Proleukin^®^ is produced in *E. coli*. Recombinant non-glycosylated IL-2 retains its full biological activity but the presence of the glycans substantially improves this protein’s solubility. Limited solubility of the non-glycosylated IL-2 is a serious problem not only because the dose of the therapeutic protein has to be increased to compensate for the lost activity but also because subsequent aggregation may cause immunogenicity [22].

In some instances both glycosylated and non-glycosylated versions of a protein may be marketed under different brand names and the various forms exhibit different pharmacokinetic and pharmacodynamic properties. Recombinant human interferon-β (rhIFN-β) is used to treat multiple sclerosis, and both a non-glycosylated (IFN-β-1b, expressed in *E. coli*) and a significantly more active glycosylated (IFN-β-1a, expressed in CHO cells) variant of rhIFN-β are used in treatment. In the case of IFN-β glycans play an important role in the protein stabilization and thus indirectly enhance its biological activity [23].

Recombinant human erythropoietin (rhEPO) produced in *Escherichia coli* is unstable to elevated temperature and tends to aggregate with time. The mammalian EPO contains about 40% carbohydrate (3 N-linked and 1 O-linked oligosaccharide chains), which makes this protein more stable and less prone to aggregate than non-glycosylated *E. coli*-derived rhEPO. Hence all marketed forms of EPO are expressed in mammalian cells [4]. There are few interesting examples of protein engineering on EPO. The first case was inspired by the observation that the content of sialic acid-containing carbohydrate in rhEPO impacted its *in vivo* activity. Sialic acid is a terminal sugar found in the glycan required for *in vivo* activity of epoetin. A glycoengineered erythropoietic molecule, darbepoetin alfa, was developed and approved for use in the treatment of some anemia cases. In this EPO construct five amino acid changes (Ala30Asn, His32Thr, Pro87Val, Trp88Asn, Pro90Thr) were introduced resulting in two additional consensus sequences for N-linked carbohydrate addition, increasing the carbohydrate content to 51% [24, 25]. Apart from increased *in vivo* activity, darbepoetin alfa has also a three-fold longer serum half-life, allowing for reduced dosing frequency [25]. In that case EPO is expressed in mammalian cells as well, but what about the production of a soluble EPO in *E. coli*? An interesting approach to improve the solubility of non-glycosylated EPO and to decrease the aggregation of *E. coli*-derived EPO was successfully tested by a group of scientists from Amgen. Three asparagine residues at positions 24, 38 and 83 were mutated to lysine residues [5]. In the native protein, these residues are the sites of N-linked glycosylation, which suggests that they should be located on the surface of the protein and should not be involved in interactions in the hydrophobic protein core. Therefore, the substitution of basic amino acids for these neutral asparagine residues is not expected to affect the protein structure, but should increase the isoelectric point of the protein and its net positive charge, decreasing its tendency to aggregate at or below neutral pH due to electrostatic interactions. Mutations resulted in a decrease in aggregation with heating or with prolonged incubation at ambient temperature, without changing the conformational stability or the receptor binding affinity of the mutant protein [5]. This approach of placing charged residues at sites where N-glycosylation occurs *in vivo* could be applied to other therapeutic proteins although it may be not always feasible. For instance IL-2 has only one glycosylation site and a single point mutation is rather insufficient to substantially improve protein solubility. Macmillan and co-workers [6] describe a quite different approach to the production of glycoproteins, in particular EPO, in *E. coli*. All three asparagine residues which are N-linked glycosylation sites were mutated to cysteine residues (Asn24Cys, Asn38Cys and Asn83Cys). This mutant was expressed in bacterial cells, refolded and subsequently subjected to a successful site specific glycosylation with β-N-glycosyl iodoacetamide [6].

### Proteolytic Protein Maturation

Eukaryotic proteins are quite often originally expressed as precursors (pro-proteins) which require further proteolytic modifications to become fully active. Moreover some protein precursors are secreted from the cell. Many of these are synthesized with an N-terminal signal peptide that targets them for secretion (pre-pro-proteins). The signal peptide is cleaved off in the endoplasmic reticulum. The most notable example here is insulin. The gene of human insulin consists of a signal peptide followed by B-chain, C-peptide and A-chain. *In vivo* the signal peptide is cleaved off after the transport of the preproinsulin to endoplasmic reticulum and C-peptide is removed by proteolysis leaving B-chain attached to A-chain via two disulfide bridges see Fig. (**1**). These extra proteolytic modifications which preproinsulin requires to become biologically active posed a great problem in the design of an expression construct for recombinant human insulin. Various engineering strategies were tested in this case [7]. Originally DNA fragments encoding respectively B-chain and A-chain were synthesized artificially and eventually expressed separately in bacterial cells. After the purification they were mixed, reduced and reoxidized [26]. This procedure appeared to be not very effective. In a different approach, which with a few modifications is still used today e.g. by Eli Lilly and Aventis, complete proinsulin is produced using a single expression construct. After the purification the proinsulin is subjected to N-terminal methionine cleavage, disulfide bridges formation and proteolytic C-peptide removal to eventually yield the biologically active recombinant human insulin [7]. This example demonstrates the versatility and potential of the *E. coli* expression system.

### Disulfide Bridges

Formation of disulfide bridges is another problem encountered when expressing eukaryotic proteins in bacterial cells. Cytoplasm is a reductive environment in which disulfide bridges cannot be formed. Eukaryotic cells possess special compartments where the proteins intended for secretion to the oxidative cell exterior are modified and this includes the formation of disulfide bridges. Human proteins which are supposed to have disulfide bridges after production in *E. coli* must be therefore reduced and reoxidized as mentioned above for the proinsulin case. This procedure adds to the complexity of the production of biopharmaceuticals and insulin is an extreme case because the disulfide bridges are supposed to connect two, otherwise separate, polypeptide chains. In a different engineering strategy a proinsulin expression construct was designed for the yeast expression system (currently used by Novo Nordisk). The construct consists of a secretion signal peptide followed by the DNA fragment encoding proinsulin, with a shortened C-peptide. This construct uses the advantage of yeast cells over prokaryotic expression systems, the fact that yeast cells are able to engineer disulfide bridges in the proteins intended for secretion. Upon secretion the signal peptide is cleaved off, thus this approach eliminates the need for two extra post processing steps in recombinant insulin production. There is no more need for N-terminal methionine cleavage and for introduction of disulfide bridges. The only remaining step is the removal of the shortened C-peptide via proteolytic cleavage.

*Escherichia coli* do have an oxidative mechanism for proteins in their periplasmic space and it was shown that they can be employed to produce human proteins which require disulfide bridges. To enhance the efficiency of the disulfide bridges formation proinsulin gene was fused by Rudolph and co-workers to the C-terminus of periplasmic disulfide oxidoreductase (DsbA) protein. This fusion allowed the production of high amounts of soluble native proinsulin in *E. coli* [9].

Secretion of the protein to the periplasmic space is also employed to produce human growth hormone (hGH) in *E. coli* [8]. There are numerous versions of this recombinant protein on the market, some produced in yeast or mammalian cells [4]. hGH molecule contains two internal disulfide bridges and, due to the absence of the signal peptide in the mature form, it does not contain the N-terminal methionine residue. The periplasmic expression is therefore the most elegant approach because it takes care of both of these post-translational modifications, providing at the same time high-yield and low cost of the *E. coli* expression system [8].

## STABILIZING PRODUCT

Protein engineering is quite often employed to stabilize recombinant human proteins produced in bacterial cells. One of the commonly introduced changes is modification of cysteine residues which influences the protein’s ability to create disulfide bridges and ultimately impacts protein stability.

Protein expressed in inclusion bodies must be solubilised and subsequently refolded to its native three dimensional structure. The refolding process is sometimes challenging especially when a protein has to recreate correct disulfide bridges. IL-2 molecule consists of three cysteine residues at positions 58, 105 and 125. In the native protein the first two of the residues form a disulfide bridge and the third one (Cys125) stays in the reduced form. It is well known that the presence of reduced cysteine residues in the oxidative environment is one of the factors contributing to protein aggregation [27]. Recombinant *E. coli*-expressed non-glycosylated IL-2 is prone to aggregation and therefore every contribution to decrease this propensity may substantially stabilize the protein. Aldesleukin (recombinant IL-2) marketed under the brand name Proleukin^®^ bears indeed the Cys125Ser mutation. It has been shown that this mutation does not alter biological activity of IL-2 [10].

Similarly IFN-β-1b molecule contains three cysteine residues at positions 17, 31 and 141, the latter two of which create a disulfide bond in the native form leaving Cys17 in a reduced state. Hence the marketed IFN-β-1b versions, Betaferon^®^ and Betaseron^®^ both contain the Cys17Ser mutation that stabilizes the product. This mutation was shown not to influence the protein biological activity [11].

Protein scientists quite often encounter situations in which a fragment of protein molecule exhibits higher instability then the remaining part. This may be caused by a variety of reasons. The most common seems to be a consecutive stretch or accumulation of hydrophobic residues in the water exposed part of the molecule. Obviously removing that part by genetic deletion may be beneficial for protein stability. This approach is quite often used in structural biology where the flexible parts with undefined three dimensional structure may become a problem e.g. in protein crystallization. Of course it is critical to ensure that the remaining part preserves 100% of the biological activity. This approach was employed in engineering a more stable variant of keratinocyte growth factor (KGF). KGF is fairly unstable, as manifested by the loss of the monomeric native protein accompanied by the accumulation of aggregated species during storage at moderate temperatures. In palifermin marketed as Biovitrum^®^, which is used in preventing chemotherapy- and radiotherapy-induced mucositis [28], 23 N-terminal amino acid residues of KGF were deleted, which was shown to substantially improve the protein stability without affecting its biological activity [12].

## MODULATING PRODUCT ACTIVITY

Protein engineering may be used not only to correct erroneous protein features resulting from expressing human protein in a foreign expression system or to improve protein stability outside of its natural environment. There are several examples of using mutagenesis to improve or modify therapeutic protein activity.

Standard pharmaceutical preparations of recombinant human insulin contain zinc ions together with a phenolic preservative. Binding of these excipients increases the stability of insulin by inducing the formation of a specific hexameric conformation. Despite the soluble state of insulin in these formulations, a delay in activity is observed. This delay has been attributed to the time required for the hexamer to dissociate into dimers and/or monomers prior to absorption from the interstitium [4]. With a detailed molecular knowledge of the dimerization process numerous analogues of human insulin were engineered with modified dimerization properties. Decreased self-association tendency minimizes the delay in the time-action. Rapid-acting insulin lispro (marketed by Eli Lilly as Humalog^®^) for instance has two point mutations in the B-chain of insulin, a proline residue (B28) and lysine residue (B29) of the wild type protein are switched to Lys(B28)Pro(B29) blocking the formation of insulin dimers and hexamers [13]. There are two more rapid-acting insulin mutants on the market: insulin aspart (Pro to Asp at position B28) and insulin glulisine (Asn to Lys at B3, Lys to Glu at B29) [14, 15]. Two long-acting insulin mutants were engineered as well; insulin glargine (Asn to Gly at A21, two extra Arg residues at positions B31 & B32) where the introduced arginine residues shift the isoelectric point of the protein making this form of insulin more soluble at acidic pH [16] and insulin detemir (Pro to Lys at B28, Lys to Pro at B29, deletion of Thr at B30) which has a greater affinity for serum albumin what increases duration of its action [17].

There are examples of using protein engineering to create a more potent therapeutic than the native human protein. Human deoxyribonuclease I (DNase I), an enzyme used to treat cystic fibrosis patients, has been engineered to more effectively degrade double-stranded DNA to lower molecular weight fragments by altering its functional mechanism from the native single-stranded nicking pathway to a much more efficient one which results in increased double-stranded scission. By introducing positively charged amino acids at DNase I positions that can interact favorably with the proximal negatively charged phosphate groups of the DNA, a hyperactive variant was created with about 35-fold higher DNA-degrading activity relative to the wild type [18]. DNase I, mainly due to the necessity of glycosylation, is expressed in mammalian cells.

In the process of engineering a more and more potent biopharmaceutical an extreme case can be imagined where the protein therapeutic does not exist naturally but is designed based on available knowledge of the targeted molecular system. Surprisingly there is an example of such a therapeutic already on the market. Interferon αcon-1, marketed by Amgen as Infergen^®^, is a recombinant non-naturally occurring type-I interferon. The 166-amino acid sequence of INF-αcon-1 was derived by scanning the sequences of several natural interferon alpha subtypes and assigning the most frequently observed residue in each corresponding position. Four additional amino acid changes were made to facilitate the molecular construction, and a corresponding DNA sequence was synthesized artificially. INF-αcon-1 differs from INF-α-2b at 20/166 amino acids (88% homology), and comparison with INF-β shows identity at over 30% of the amino acid positions [19]. The antiviral, antiproliferative, NK cell activation activity, cytokine induction, and interferon-stimulated gene-induction activity of consensus interferon has been compared with naturally occurring type I interferons. In all of these comparisons, consensus interferon had a higher activity when compared with IFN-α-2a and IFN-α-2b [20].

## GENETIC FUSIONS

Genetic engineering allows not only an exchange of one or several amino acid residues, but therapeutic proteins may be genetically fused to other proteins and expressed together as one polypeptide chain. Contrary to chemical conjugation this approach ensures a very elegant and biocompatible way of coupling biopharmaceuticals to other proteins. Protein fusions are commonly used in biochemistry to facilitate protein purification and/or to enhance target protein solubility by attaching it to a larger soluble fusion partner. There are several examples of genetic fusions applied in the engineering of therapeutic proteins and they serve a whole variety of purposes.

One of the major problems with protein therapeutics is their short half-life in the circulation. There are currently several drugs in development which explore the idea of attaching a protein of interest to human albumin [29]. Albumin is a naturally occurring, widely distributed and enzymatically inert serum protein with a long half-life. It serves as a carrier of blood substances. Albinterferon α-2b (alb-IFN), a recombinant polypeptide composed of IFN-α-2b genetically fused to human albumin, has an extended half-life and early evidence indicates that it is efficacious and well tolerated [29, 30]. Other examples of therapeutic proteins fused to albumin, which are currently in development, include insulin [31], growth hormone [32], IL-2 [33] and B-type natriuretic peptide [34]. In all these cases albumin fusion offers increased *in vivo* half-life and increased solubility and stability but at the same time a risk of immunogenicity.

One of the recent strategies to overcome poor oral absorption of protein drugs is by genetic fusion with transferrin (Tf). Tf is a serum protein that transports iron to cells expressing Tf receptor (TfR) on the plasma membrane through TfR-mediated endocytosis [35]. An expression construct was engineered by fusing human cDNA encoding granulocyte colony-stimulating factor (G-CSF) to the gene of transferrin. *In vitro* and *in vivo* studies demonstrated that the purified G-CSF-Tf fusion protein possesses the G-CSF activity and is absorbed via a TfR-mediated process in the gastrointestinal tract [36]. Similar results have been produced for the transferrin fusion with hGH [37]. Although these results are very promising, due to the large size of Tf molecule (about 80 kDa), internal disulfide bridge and glycosylation, this approach is suitable for eukaryotic expression systems. Similarly proteins fused to human albumin are in most cases produced in *Saccharomyces cerevisiae*.

## IMPORTANCE OF THE “CLEAN” CONSTRUCT

A discussion of the various expression vectors goes beyond the scope of this article, however there are certain elements of the expression vector design which are extremely important for therapeutic protein production. There are numerous vectors available commercially; many of them offer easiness of cloning, high-throughput options or few tags which are automatically attached to the final product to facilitate subsequent protein purification and/or immunological detection. The literature is abundant in data which were produced with protein constructs genetically attached to long tags which contain a lot of extra amino acid residues that may substantially influence results. When designing an expression construct for a protein which is intended to be used pharmaceutically, one has to take into account each and every one of the extra residues introduced to the final protein product via cloning. Even a single amino acid residue changed or added to the protein sequence may not only alter the biological activity *in vivo* but may cause immunogenicity, which is one of the major problems in therapeutic protein development.

Human insulin differs from porcine insulin by a single amino acid residue (Thr instead of Ala) at the C-terminal residue of the B-chain. In spite of the fact that the protein activity is not affected by this small difference [38], a technology was develop to “humanize” the porcine insulin [39] and this humanized insulin was the first large scale source of this therapeutic with the sequence identical to human insulin. Difficulties obtaining sufficient supplies of porcine pancreata and recent concerns over transmissible spongiform encephalopathies associated with the use of animal-derived materials were the reasons for discontinuing the production of this type of insulin.

Polypeptide chain synthesized by a ribosome always starts with a methionine residue. It has been mentioned above that quite often human proteins are preceded with a signal peptide which is subsequently cleaved off in the mature protein; as a consequence mature human proteins do not necessarily contain methionine at their N-terminus. When designing a construct for expression of such a protein in *E. coli*, it is therefore necessary to include an extra methionine residue at the N-terminus. Many approved protein therapeutics are expressed with this extra residue. In some cases the methionine is cleaved off in the protein post-processing, and this is the case for the recombinant human insulin [7] but there are numerous cases where, to simplify the production process, the proteins are left with this modification. The examples include granulocyte colony-stimulating factor (G-CSF, filgrastim) [40], stem cell factor (SCF, ancestim) [41] and IL-1 receptor antagonist (IL-1Ra, anakinra) [42].

## CONCLUSIONS

Protein engineering is a very powerful tool in the development of biopharmaceuticals. It has been successfully employed in improving various features of therapeutic proteins, not only to overcome missing posttranslational modifications, but also to improve protein stability and/or modulate its function (Table **1**). Thus, increasing knowledge on molecular basis of various diseases may be used here very practically. However the care must be taken to avoid immunogenicity or other unwanted side effects.

## Figures and Tables

**Fig. (1):**
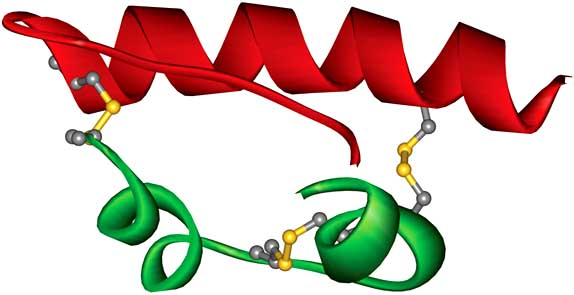
In spite of being a small molecule, insulin posed a great challenge for expression attempts in *E. coli* due to its complex post-translational modifications. Mature insulin consists of two separate polypeptide chains, **B** (red) and **A** (green), interconnected via two disulfide bridges (ball and stick representation: carbon atoms in grey, sulfur atoms in yellow). The molecule contains also a third disulfide bridge which is internal for the chain **A**. The image was prepared from the 1znj PDB file using WebLab Viewer software.

**Table 1:** Examples of Strategies for Engineering of Therapeutic Proteins

Issue Addressed		Engineering Strategy	Example Reference
Lack of posttranslational modifications in *E. coli*	Glycosylation	Expression without the glycosylation (only if the biological activity is not impaired)	IL-2 [3], IFN-β-1b [4]
		Mutation of residues on the surface to create more soluble protein	EPO [5]
		Mutation of glycosylation sites to cysteines to allow subsequent glycosylation *in vitro*	EPO [6]
	Proteolytic maturation	Expression in two separate strains or cleavage of the precursor *in vitro*	insulin [7]
	Disulfide bridge formation	Expression in the periplasmic space	hGH [8], proinsulin [9]
Protein stability		Decreasing number of free cysteine residues by mutation to alanine	IL-2 [10], IFN-β-1b [11]
		Deletion of the hydrophobic region	KGF [12]
Modulation of protein activity		Design of the rapid-acting or long-acting protein version	insulin [13-17]
		Enhanced activity by improved affinity to the target molecule	DNaseI [18]
		Design of protein consisting of a consensus sequence	IFN-α-con [19, 20]
